# Proactive and Reactive Processes in the Medial Frontal Cortex: An Electrophysiological Study

**DOI:** 10.1371/journal.pone.0084351

**Published:** 2014-01-03

**Authors:** Flavio T. P. Oliveira, Clayton Hickey, John J. McDonald

**Affiliations:** 1 Department of Psychology and Helen Wills Neuroscience Institute, University of California, Berkeley, United States of America; 2 VU University Amsterdam, Amsterdam, The Netherlands; 3 Department of Psychology, Simon Fraser University, Burnaby, British Columbia, Canada; Cardiff University, United Kingdom

## Abstract

The posterior medial frontal cortex (pMFC) is known to be involved in adaptive goal-directed behavior, but its specific function is not yet clear. Most theories have proposed that the pMFC monitors performance in a reactive manner only, but it is possible that the pMFC also contributes to performance monitoring in a proactive manner. To date, the evidence for proactive pMFC activity is equivocal. Here, we investigated pMFC activity before, during and after the performance of a challenging motor task. Participants navigated a cursor through narrow and wide mazes in randomly intermixed trials. On each trial, participants saw previews of the actual maze display prior to gaining control of the cursor. Event-related potentials (ERPs) to the preview displays were compared to ERPs elicited by no-go signals and errors. Compared to the wider maze, the preview display for the more challenging narrow maze elicited a medial-frontal negativity (MFN) similar to the ERP components elicited by no-go signals and errors. Like these known ERP components, the preview-elicited MFN appeared to be generated from a source in pMFC. This is consistent with the hypothesis that the pMFC participates in adaptive behavior whenever there is a need for increased effort to maintain successful task performance.

## Introduction

Converging lines of evidence suggest that the posterior medial frontal cortex (pMFC), an area that encompasses the anterior cingulate cortex (ACC) and the pre-supplementary motor area (pre-SMA), plays a critical role in adaptive, goal-directed behavior [1]. This view is supported by studies of psychiatric disorders affecting pMFC function [2] and by studies with non-human species [3]–[7] and human patients with lesions to pMFC [8]–[10], showing that pMFC dysfunction leads to deficits in voluntary goal-directed actions. However, the precise functions of the pMFC are not yet clear.

The most prominent theories link activity within this cortical region to the monitoring of task performance. For example, the conflict-monitoring hypothesis proposes that pMFC detects response conflict occurring when two competing responses are co-activated and signals to pre-frontal cortex the need for increased cognitive control [11]–[16]. In contrast, the error-detection and reinforcement-learning hypotheses propose that the pMFC is part of a reward-prediction system that is activated whenever performance is worse than expected [17]–[20]. Both of these general views suggest that the pMFC responds in a *reactive* manner only – that is, it monitors performance online or it contributes to learning after the task has been performed.

Researchers have begun to challenge the notion that the pMFC monitors performance exclusively in this kind of reactive manner, suggesting instead that the pMFC can act proactively to alter performance on an upcoming task [21]–[28]. Such anticipatory pMFC activity is consistent with two recent hypotheses of pMFC function. According to one of these, the degree to which pMFC is activated is directly proportional to the likelihood of making an error [23]. Critically, this *error-likelihood hypothesis* suggests that increased pMFC activity can be triggered before, during, or after task performance – as long as the eliciting event signals a high likelihood of error. Empirical evidence for the error-likelihood hypothesis has been mixed, however: the original error-likelihood study reported fMRI results consistent with the hypothesis [23], but a series of subsequent experiments detected no anticipatory pMFC activity in response to error-likelihood cues [29].

An alternative hypothesis posits that the pMFC is sensitive to cues that signal situations that demand increases in cognitive or physical effort [1], [30]–[32]. According to this *effort-allocation hypothesis*, pMFC is involved in representing and updating action-outcome contingencies based on the amount of effort required by the task and the potential benefit that these actions might generate. The pMFC might be further involved in allocating the necessary effort to select and produce actions that lead to favorable consequences [31]–[33]. Within this framework, errors, negative feedback, and response conflict are considered instances of a broader class of events that trigger pMFC involvement, namely events that signal the need for increased effort to update action-outcome contingencies or to select and produce actions with favorable consequences.

According to the effort-allocation hypothesis, pMFC is responsive only to information that can be used to alter behavior or action-outcome associations, and this defines an important distinction between the effort allocation hypothesis and the error-likelihood hypothesis. Cues indicating error-likelihood do not necessarily carry this type of information: a signal indicating increased error likelihood need not provide information about how to improve performance. The pMFC may become active only when circumstances are such that a strategic response to information about error likelihood is possible, suggesting a link with effort allocation rather than pure error likelihood, and this could underlie mixed results in tests of the error-likelihood hypothesis [29].

The present study used event-related potentials (ERPs) to determine whether the pMFC is active prior to, as well as during, performance of a challenging motor task. An important aspect of this motor task was that it provided information that could directly guide the selection of action and lead to preparatory adjustments before participants could begin to solve the task. We predicted that under these conditions the pMFC would contribute to behavioral adaptation proactively as well as reactively. To test this prediction, we developed a virtual-maze task in which observers previewed a maze prior to performing a maze-navigation task. This task required that the mouse cursor be kept on the maze path, and we varied the difficulty of the task by randomly intermixing narrow and wide mazes. The preview of the to-be-navigated mazes informed participants whether errors would be more or less likely and enabled participants to prepare for successful completion of the impending task.

The high temporal resolution of ERPs allowed us to measure separate ERP components in response to the preview displays, subsequent Go and No-Go signals, and the eventual successful and unsuccessful maze navigations (i.e., correct responses and errors). Critically, this enabled direct comparisons of the proactive ERP activity elicited by narrow preview displays and the well-known reactive ERP activities associated with response conflict stemming from the No-Go signals [34]–[36] and with response errors [37], [38]. Such preparatory ERP activity could not be attributed to conflict between competing task sets because participants performed only one task throughout the experiment.

## Materials and Methods

### Ethics Statement

Experimental procedures were approved by the Simon Fraser University research ethics board.

### Participants

Sixteen healthy university students participated in the study. Data from two participants were excluded from all analyses because of excessive noise in the electroencephalogram (EEG). The remaining 14 participants (6 women, 1 left-handed, mean age: 21.6 years, range: 18–26 years) provided written informed consent prior to the start of the experiment and received course credit for their participation.

### Apparatus

The experiment was conducted in a sound attenuated and electrically shielded chamber that contained a 19-in CRT monitor with the screen resolution set to 800×600 pixels. Participants sat in an adjustable chair and viewed the monitor from a distance of 65 cm. A computer running Presentation (Neurobehavioral Systems Inc., Albany, CA, USA) controlled stimulus presentation and registered the participants' mouse movements. A second computer running custom software (Acquire) controlled EEG acquisition. The acquisition computer housed a 64-channel A-to-D board (PCI 6071e, National Instruments, Austin, TX, USA) that was connected to an EEG amplifier system with high input impedance (SA Instrumentation, San Diego, CA, USA).

### Stimuli and Procedures

**Figure 1:**
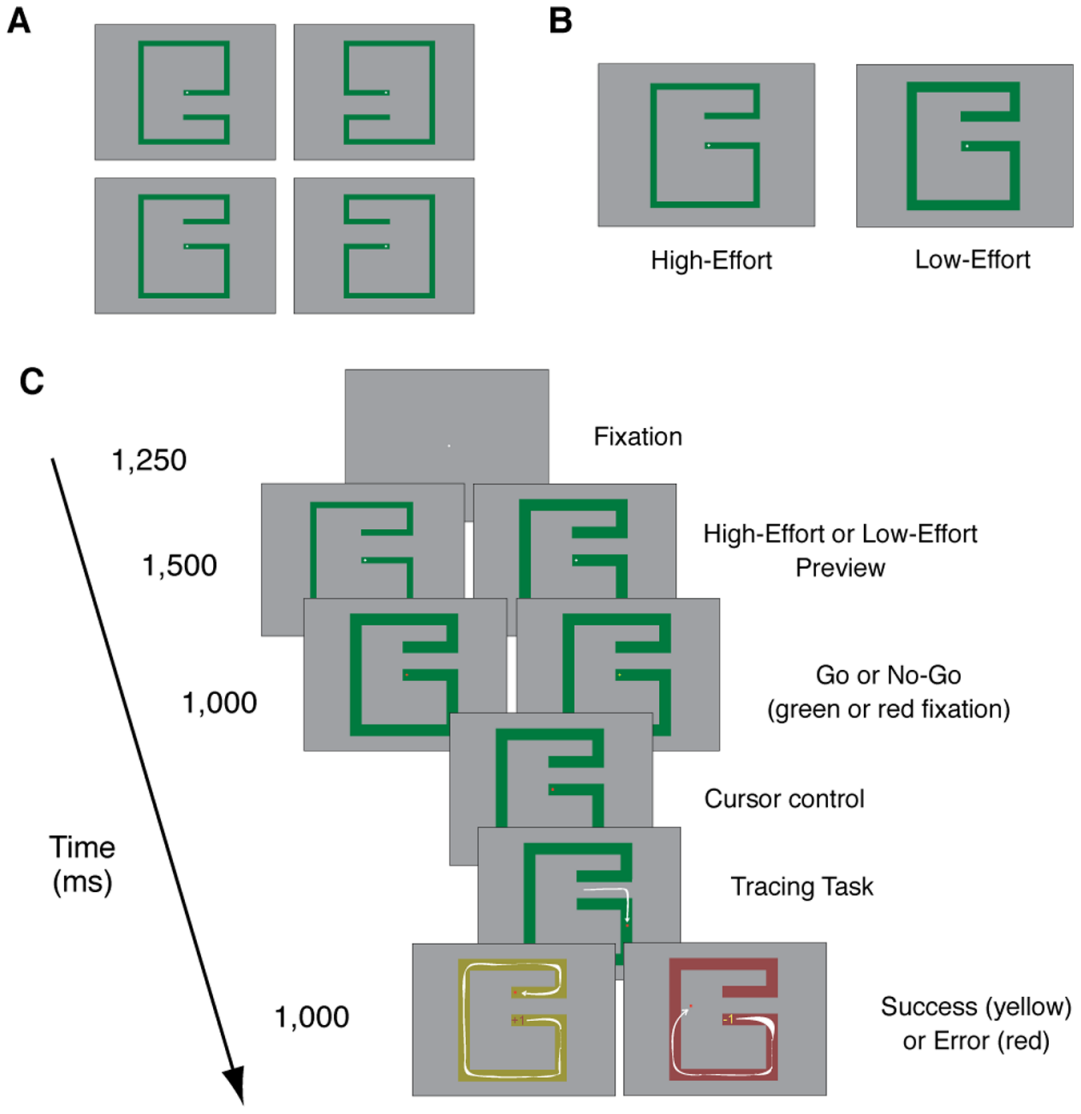
Stimuli and Task. **(A)** The four different spatial configurations for the maze. **(B)** Examples of Low-Effort and High-Effort mazes. **(C)** Schematic representation of the events on each trial. The trial started with a white central fixation presented for 1,250 ms. Following, one of the eight possible mazes was added to the fixation display. The presentation of the maze provided participants with a perceptual cue about how much effort the task was going to require. The fixation cross then changed color to red or light green to indicate a No-Go or Go trial, respectively. On Go trials, the fixation cross was replaced by a square cursor after 1,000 ms and the participants gained cursor control. Upon successful completion of the task, the maze turned yellow. In case of errors or time-outs the maze turned red.

Participants navigated a cursor through a single-path maze using a standard computer mouse with the right hand (Figure 1). Each trial started with the presentation of a white fixation cross (5 mm×5 mm) at the center of the screen on a grey background for 1,250 ms. Following this fixation display, a preview display containing the fixation cross and one of eight possible mazes was presented. The eight possible mazes were green and differed according to four spatial configurations (Figure 1A) and two path widths (Figure 1B). The narrow (9 mm width) and wide (18 mm width) mazes were designed to require more or less effort to navigate successfully, thereby representing High-Effort and Low-Effort conditions, respectively (Figure 1B). Low-effort and high-effort mazes were randomly assigned to each trial and occurred with equal frequency. The preview of the low-effort and high-effort mazes provided participants with advanced information about the amount of effort needed to complete each maze and also with information (e.g., direction and width of the path) that allowed them to prepare for the tracing task. After 1,500 ms, the fixation cross changed color to either green or red (randomly intermixed with equal frequency) to indicate a Go or a No-Go trial, respectively. The red or green fixation crosses remained on the screen for 1,000 ms. In the case of a No-Go trial, the participants were then presented again with a solitary white fixation cross as the start of the following trial. In the case of a Go trial, the green fixation cross was replaced by a white square cursor. Participants gained control of this cursor and could begin moving the cursor along the maze path. Trials were considered successful if the entire maze was navigated without this cursor leaving the path. If the cursor left the maze path, the maze changed color to red immediately for 1,000 ms and central feedback indicated the loss of one point. If the maze was successfully navigated, the maze changed color to yellow for 1,000 ms and central feedback indicated the gain of one point. The Go trial ended after the yellow (positive) or red (negative) feedback display (Figure 1C).

On the first trial, participants had a limit of 13 seconds to complete the task, after which the maze turned red representing a timed-out trial and central feedback indicated the loss of one point. The time limit varied from trial to trial according to an adaptive procedure. After each unsuccessful trial, the time limit was increased by 250 ms and after each successful trial the time limit was decreased by 250 ms. All displays and colored stimuli were physically equiluminant at 6.00 cd/m^2^. To increase motivation, participants were told that the experiment was in fact a game in which they earned points for each successfully completed task and lost points for time-outs and errors. At the end of each block participants received feedback about their accumulated score in point units, along with a 'scoreboard' that presented scores earned by earlier participants in the experiment. The results displayed in this scoreboard were fabricated and were included in the design only to motivate participant effort. Each block consisted of 16 trials. The full experimental session was composed of 30 blocks.

### Behavioral measures and analysis

As indirect measures of the effort needed to complete the task, we calculated the percentage of trials on which participants successfully navigated the maze (correct), went off the maze path (incorrect), or did not complete the maze in the allotted time (time-out), separated by effort condition. We further calculated the amount of time it took for participants to successfully complete the Low-Effort and High-Effort mazes (herein termed *completion time*).

We were also interested in obtaining a measure of the behavioral adjustments made in response to increased recruitment of control. In performance-monitoring studies, a commonly used measure of monitoring is post-error or post-conflict slowing of reaction time (RTs; [39], [40]). To investigate anticipatory behavioral adjustments on task performance in the maze task, we compared RTs in the High-Effort and Low-Effort conditions. This allowed us to assess the proactive recruitment of control and behavioral adjustments generated by the presentation of the preview displays indicating the amount of effort necessary to solve the task (i.e., the mazes). Here, RTs were measured as the time it took for participants to start moving the mouse after they gained control of the cursor. All behavioral results were analyzed by one-tailed paired permutation tests based on all possible (2^14^) permutations of the data.

### Electrophysiological recording and pre-processing

We recorded EEG from 63 tin electrodes attached to an elastic-fabric cap (Electro-Cap International, Inc). All but 5 of these electrodes were positioned according to the 10-10 system (FPz, FP1, FP2, AF3, AF4, Fz, F1, F2, F3, F4, F5, F6, F7, F8, FCz, FC1, FC2, FC3, FC4, FC5, FC6, Cz, C1, C2, C3, C4, C5, C6, T7, T8, CPz, CP1, CP2, CP3, CP4, CP5, CP6, Pz, P1, P2, P3, P4, P5, P6, P7, P8, P9, P10, POz, PO3, PO4, PO7, PO8, Oz, O1, O2, Iz, and M1). The remaining 5 electrodes were positioned inferior to the standard row of occipital electrodes. Horizontal electrooculographic (EOG) signals were recorded bipolarly using electrodes positioned at the external canthi. All EEG electrodes were referenced to an electrode placed on the right mastoid (M2). Electrode impedances were kept below 10 kΩ. EEG signals were amplified by a gain of 20,000 and a band pass of 0.1-100 Hz, digitized at 500 Hz and stored on a computer for offline averaging. Offline analysis was performed with EEGLAB [41]. The data were down-sampled to 250 Hz, re-referenced to an average reference, epoched and filtered offline with a bandpass of 0.5–30 Hz. We then used blind source separation based on second order blind identification (SOBI) to remove ocular artifacts [42] and based on canonical correlational analysis (CCA) to remove electromyographic (EMG) artifacts from the data [43]. This was followed by an automated procedure to exclude trials with activity deviating by 6 standard deviations or more from the probability distribution of all trials for each participant and also trials with activity greater than 100 µV or smaller than -100 µV. Excluded trials accounted for an average of 4.7% of the total trials.

### ERP analysis

After pre-processing the data, we extracted ERPs by averaging EEG epochs separately for 6 conditions of interest. In the first analysis we compared ERPs elicited by the initial presentation of the Low-Effort and High-Effort mazes (preview displays). We further looked at the correlation between the amplitude of the ERP component elicited in response to the preview displays and completion and reaction times. In the second analysis we compared ERPs elicited by the presentation of the Go and No-Go signals, and in the third analysis we compared ERPs elicited by the successful completion of the task and by errors. All ERPs were baseline corrected using the average amplitude of the 200-ms period preceding onset of the relevant event of interest (preview displays, Go/No-Go signals and successful completion or errors). All statistical comparisons were performed on the mean amplitude of the waveforms for the FCz electrode during 80-ms time windows centered on the time of peak amplitude of the components of interest (see Results). The comparisons were performed through one-tailed paired permutation tests based on all possible permutations of the data (214).

### Source analysis

We used a two-step procedure for source analysis. First we used BESA 5.3 software (Megis software) to generate a distributed linear solution based on a Classical LORETA Analysis Recursively Applied (CLARA) model [44]. CLARA is performed by applying the Low Resolution Electromagnetic Tomography (LORETA; [45]) algorithm iteratively, resulting in a reduction of the source space. The CLARA model we used was based on four iterations of LORETA with a SVD cutoff of.005% on the first run and.01% on the following iterations. We plotted the activation foci from the CLARA model on the Colin brain—a high resolution average of 27 MRI scans of one brain [46]. On the second step, we created a dipole model using the coordinates of each source foci found in the CLARA analysis. We fixed dipoles to the coordinates of maximal CLARA activity and fitted the orientation of the dipoles for the time window of interest. This two-step procedure allowed us to calculate the amount of variance explained by a dipole model informed by the CLARA analysis. This provided a data-driven method to estimate the number and location of dipoles. Whereas minimum-norm based solutions such as CLARA require no a priori assumption of the number and location of activity foci, dipole models are highly sensitive to such assumptions [47].

## Results

### Behavior

**Figure 2:**
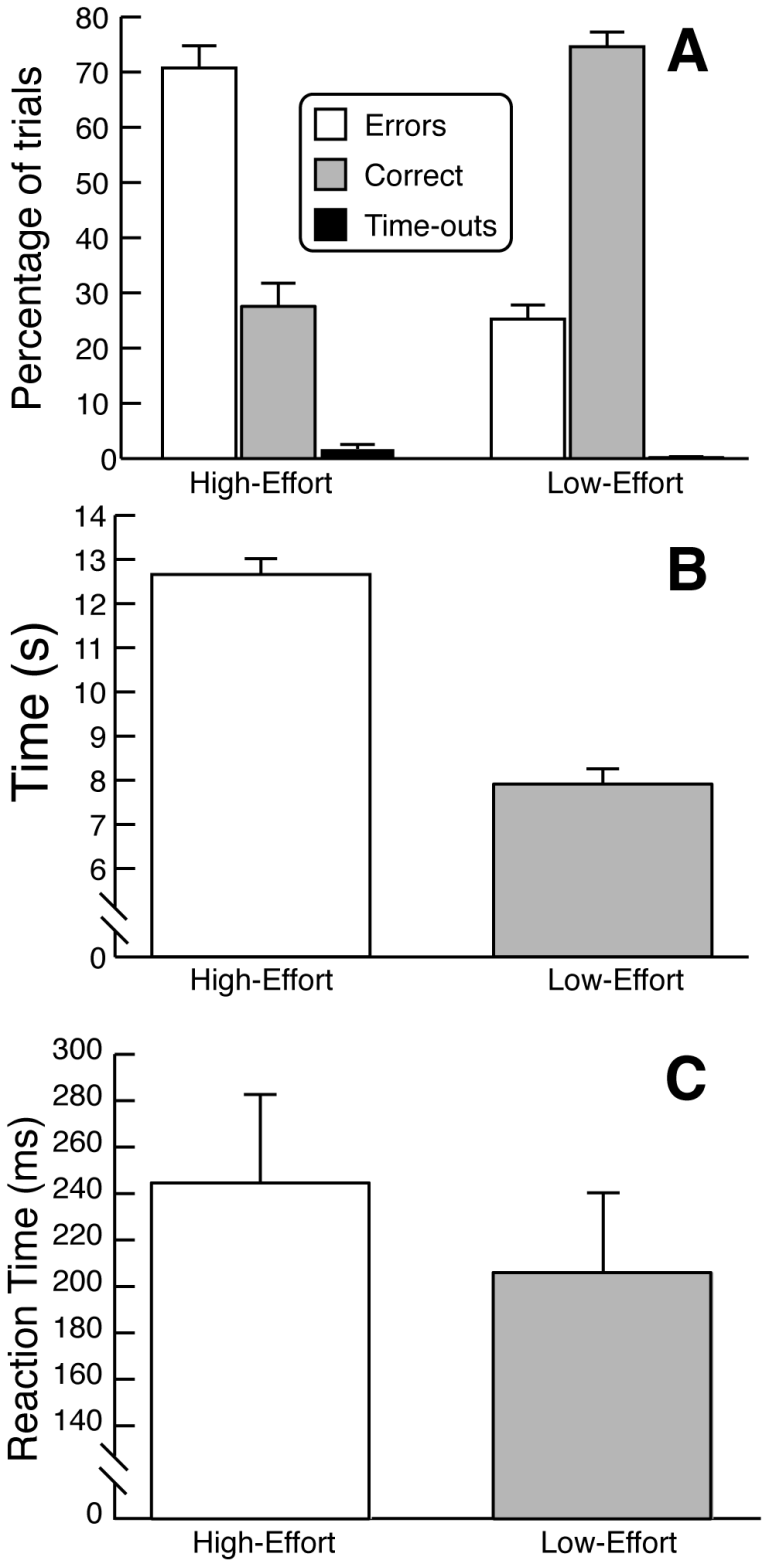
Behavioral Results. **(A)** Mean percentage of trials that were successful or ended in an error or time-out. **(B)** Mean task completion time in seconds for the two effort conditions. **(C)** Mean reaction time in milliseconds for the two different effort conditions.

The behavioral results showed that the High-Effort maze was considerably more difficult to navigate than the Low-Effort maze (Figure 2). Participants made errors in a larger percentage of trials in the High-Effort condition (71%) than in the Low-Effort condition (25%, p = .0001, Figure 2A), committed more time-outs (High-Effort: 1.7% of trials; Low-Effort: 0.2% of trials, p = .016, Figure 2A) and took more time to complete the task (High-Effort: 12.7 seconds; Low-Effort: 7.9 seconds; p = .0001, Figure 2B). The results also showed that participants adjusted to the different control requirements in a proactive, anticipatory manner by increasing preparation time. RTs were significantly longer for the High-Effort (245 ms) than the Low-Effort (206 ms; p = .0002, Figure 2C) conditions.

### Electrophysiology

**Figure 3:**
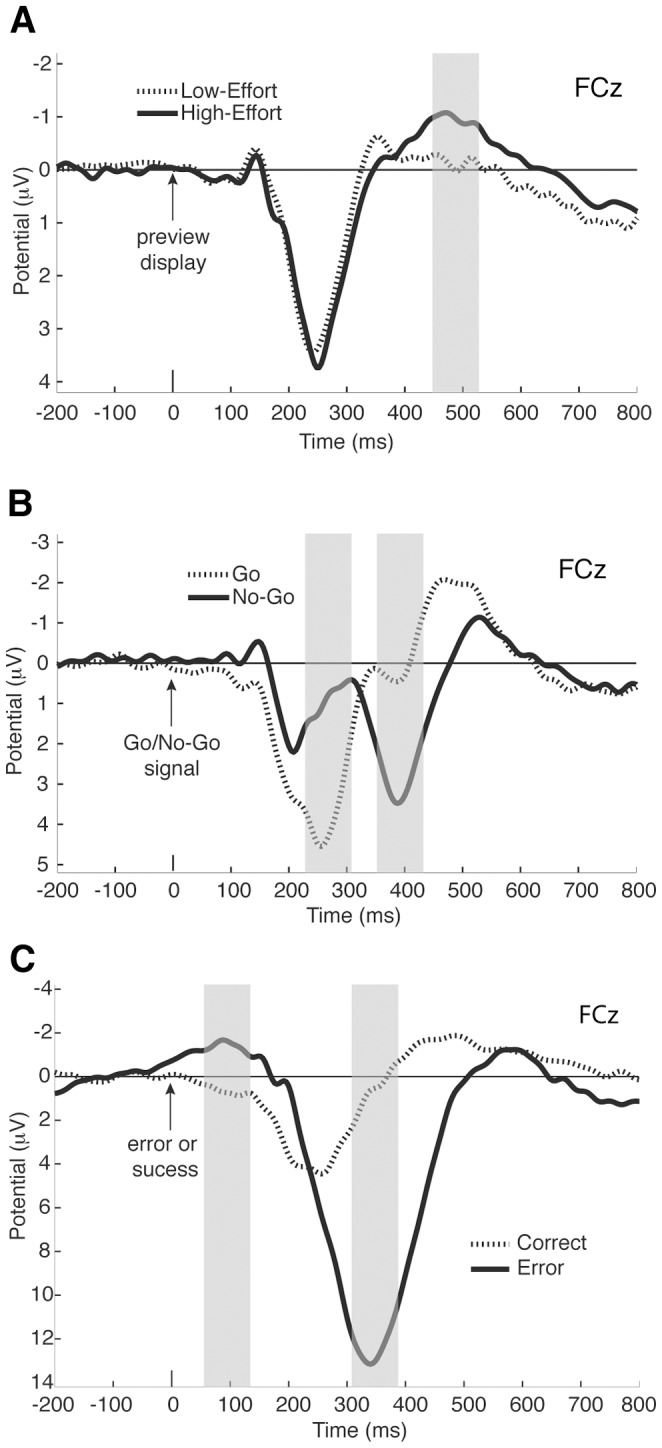
Grand-average ERP waveforms recorded at fronto-central electrode FCz. Negative voltages are plotted upward by convention. Shaded regions represent the time windows used for statistical analyses (**A**) ERPs elicited by the Low-Effort and High-Effort preview displays. (**B**) ERPs elicited by Go and No-Go signals. (**C**) ERPs elicited by errors and correct responses.

**Figure 4:**
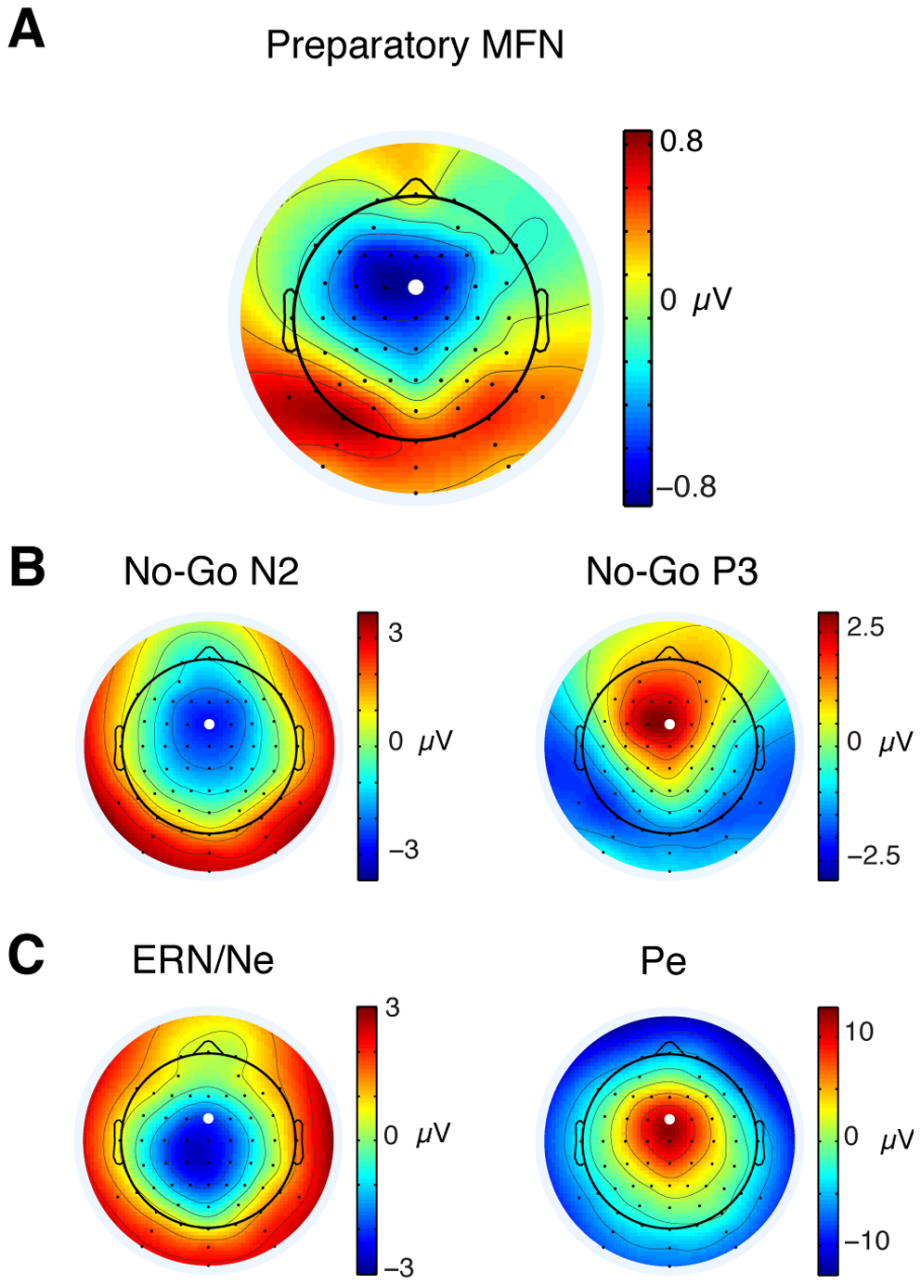
Topographical maps. Black dots represent locations of electrodes, and the white dot represents the location of the fronto-central electrode where the ERPs were plotted. (**A**) Preparatory medial frontal negativity time-locked to the onset of the preview display (448–528 ms post-stimulus). (**B**) No-Go N2 (left) and P3 (right) time-locked to the onset of the Go/No-Go signal (228–308 and 352–432 ms post-stimulus, respectively). (**C**) Error related negativity (ERN/Ne, left) and positivity (Pe; right) time-locked to the end-of-trial event (error or successful completion of the maze; 56–136 and 308–388 ms post-stimulus, respectively).

In our main contrast of interest, we compared the ERPs elicited by the onset of the Low-Effort and High-Effort preview displays. Figure 3A shows the ERP waveforms for the two conditions at electrode FCz. A pronounced difference between the two conditions emerged at around 400 ms, peaked at around 480 ms and lasted until 800 ms after the onset of the mazes. This difference led to a reliably larger negative-going deflection in the ERP waveform for the High-Effort condition compared to the Low-Effort condition during the time window we analyzed it (448–528 ms, Figure 3A; p = .0016). This negativity was maximal at fronto-central electrode sites (Figure 4A), which is consistent with previously reported ERP components attributed to pMFC activity [36], [38], [48]. We found no significant correlation between behavioral measures and the amplitude of the ERP component elicited in response to the preview displays.

In the second contrast of interest, we compared ERPs elicited by the onset of the Go and No-Go signals. Figure 3B shows the ERP waveforms for the two conditions at electrode FCz. In the ERP waveforms elicited by both the No-Go and the Go signals, a positive-going deflection emerged starting at around 150 ms after the onset of the signals. This positive-going deflection peaked at around 250 ms in the Go waveform. In the No-Go waveform, a negative-going deflection started emerging at around 200 ms and peaked at around 300 ms. This deflection is consistent with the No-Go N2 [36], [49], [50] and was absent from the Go waveform, thus generating a reliable difference between the two conditions during the time window we analyzed it (228–308 ms; p = .00006). Consistent with previous reports of the No-Go N2, this negative-going peak was maximal at fronto-central electrode sites (Figure 4B, left; [36], [50]). After the peak of the N2, a positive-going deflection emerged in the No-Go waveform, which generated a reliable difference between the two conditions during the time window analyzed (352–432 ms; p = .00006). This difference was consistent with a No-Go P3 [49], [50] and was also maximal at fronto-central electrode sites (Figure 4B, right).

In the third contrast of interest, we compared ERPs elicited by errors and correct task performance. We time-locked the ERPs to the moment the errors occurred (i.e., the cursor left the maze path) and the moment the task was successfully completed (i.e., the cursor reached the end of the maze path). These times were also marked by a change of maze color to indicate the end of a correct trial or an error. Figure 3C shows the ERP waveforms for the two conditions at electrode FCz. In the error waveform, an early negative-going deflection emerged peaking at around 90 ms after the onset of the errors. This negative-going deflection was absent from correct trials generating a reliable difference between the two conditions during the time window we analyzed it (56–136 ms; p = .0019). This negativity is consistent with previous reports of the error negativity (Ne) or error-related negativity (ERN) [17], [18]. Analysis of the scalp topography of this negativity revealed that it was maximal at central electrode sites, slightly posterior to what we found for the effort-related and No-Go-related components (Figure 4C, left). Previous reports have frequently found the ERN/Ne to be maximal at fronto-central sites. The central maximum that we found could be partly due to an overlap with sensory potentials related to the different colors of the stimuli. It is nevertheless consistent with several studies investigating the ERN/Ne [48], [51], [52]. After the ERN/Ne, a large positive-going deflection emerged in the error waveform peaking at around 350 ms after the onset of the error. This positivity is consistent with previous reports of the error positivity (Pe; [17]) and was reliably larger than the positivity for the correct waveform during the time window we analyzed it (308–388 ms, Figure 3C, p = .00006). Analysis of the scalp topography of this positivity revealed that it was maximal at fronto-central electrode sites (Figure 4C, right), which is consistent with previous reports of the Pe [53], [54], [55], but could also be related to a functionally distinct component observed earlier and with a more anterior topography compared to the Pe [48], [56].

### Neural source estimations

**Figure 5:**
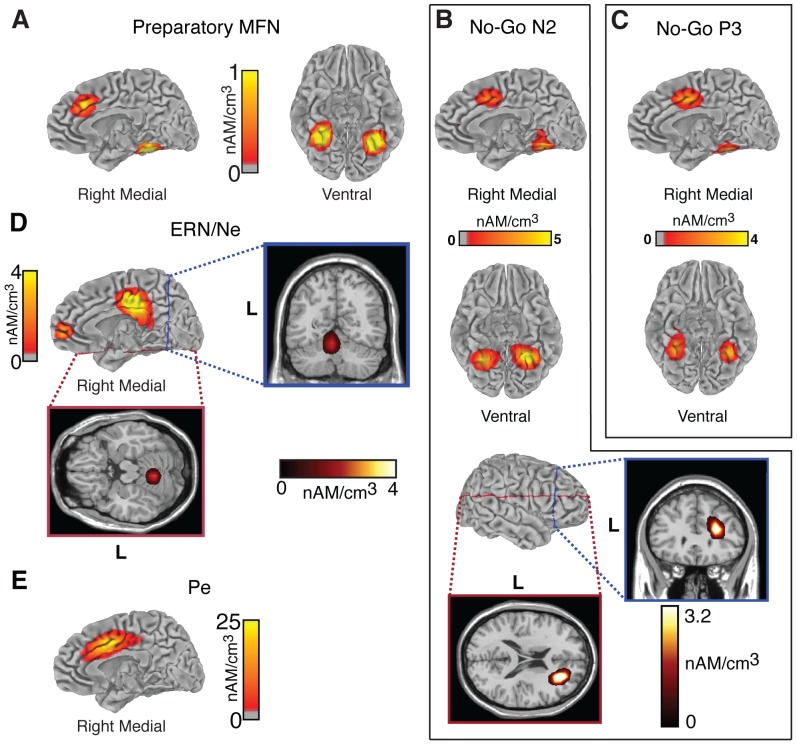
Estimated distributed source activity underlying the ERPs of interest. Source estimations are plotted on the medial and ventral surfaces of the brain in real space (i.e., image left  =  anatomical left). (**A**) Sources of the preparatory MFN, measured 488 ms after the onset of the preview display. (**B and C**) Sources of the No-Go N2 and P3, measured 268 ms and 392 ms after the onset of the Go/No-Go signal. (**D and E**) Sources of the error related negativity and error positivity (ERN/Ne and Pe), measured 96 ms and 348 ms after the onset of the end-of-trial response event.

Neural sources were estimated using CLARA at the time of peak activation for each of the five contrasts of interest. Consistent with our predictions, the CLARA analysis showed increased activation in the pMFC in response to the presentation of the High-Effort preview display compared to the presentation of the Low-Effort preview display. This activity was estimated to be in the dorsal-rostral ACC and was accompanied by increased activation in bilateral fusiform gyrus (Figure 5A). The dipole model we created based on these activation foci had 3 dipoles: one in the ACC and one in each hemisphere's fusiform gyrus. We fitted the orientation of these dipoles during the same time window in which we performed the ERP analysis (448–528 ms). The dipole model showed a good fit, accounting for 97.1% of the variance in this time window. An analysis of the contribution of each dipole to the overall model confirmed that the pMFC source accounted primarily for the medial frontal negativity, while the two sources in fusiform gyrus accounted for the positivities over the posterior scalp.

We followed similar procedures with the source analysis for the two ERP components identified for the No-Go minus Go contrast. Similar to what we found for the High-Effort minus Low-Effort contrast, the CLARA analysis estimated that the dorsal ACC and bilateral fusiform gyri were involved in the generation of the No-Go N2 (Figure 5B). In addition to those sources, a focus of activity was also estimated in the right inferior frontal sulcus, extending into dorsal- and ventral-lateral prefrontal cortices. This is consistent with previous studies implicating the right lateral prefrontal cortex in inhibitory control [57]–[60]. The dipole model we created based on these activation foci had four dipoles: one in ACC, one in each hemisphere's fusiform gyrus and one in right inferior frontal sulcus. We fitted the orientation of these dipoles during the same time window in which we performed the ERP analysis (228–308 ms). The dipole model showed a good fit, accounting for 98.0% of the variance during this time window. The CLARA-estimated sources for the No-Go P3 were similar to those of the No-Go N2 but without the right lateral prefrontal focus (Figure 5C). The dipole model with three dipoles (one in ACC and one in each hemisphere's fusiform gyrus) accounted for 98.1% of the variance during the 352–432 ms time window.

The CLARA analysis revealed three neural sources of the ERN/Ne (Figure 5D). The first source was located rostral to the genu of the corpus callosum in the ACC and extended into the frontal pole. The second source was located in posterior cingulate cortex and the third source was estimated to be in the cerebellum. We created a dipole model based on these three activation foci and fitted the orientation of these dipoles during the same time window in which we performed the ERP analysis (56–136 ms). This model accounted for 96.2% of the variance. We then removed the cerebellar dipole and fitted the model with only two dipoles: one in ACC and one in posterior cingulate cortex. This two-dipole model maintained a good fit accounting for 94.8% of the variance.

The CLARA model for the Pe contained a single source, which was located in the ACC and extended into posterior cingulate cortex (Figure 5E). The dipole model based on this ACC source accounted for 98.0% of the variance during the time-window of interest (308–388 ms).

The foci in ACC and posterior cingulate are consistent with previous studies performing source estimation for the ERN/Ne [38], [53], [61] and Pe [56].

## Discussion

In the present study we developed a new paradigm to investigate the proactive involvement of pMFC in effort-based behavioral adjustments. Our data showed that the presentation of the High-Effort maze elicited a *preparatory medial frontal negativity* (MFN) that peaked at around 480 ms after the onset of the maze and had a pMFC source. This finding adds to the growing body of literature showing pMFC activity elicited proactively in anticipation of task performance [21], [23], [24], [26], [30].

### Effort allocation and error likelihood

The preparatory MFN observed here is consistent with both the effort-allocation and error-likelihood accounts of the pMFC: the High-Effort preview display predicted increased error-likelihood, which could also be seen as a negative prediction error if participants generated the expectation of a low-effort trial. Such an expectation would not have been based on a preponderance of low-effort trials because low- and high-effort trials were equally likely and randomly intermixed. However, participants likely preferred low-effort trials and thus may have come to anticipate them. The preview display also provided participants with information about the effort necessary to successfully navigate the maze. This latter feature of the design might be crucial. Extant studies that have failed to find increased pMFC activity in response to high error-likelihood cues provided no information that could be used proactively to improve performance (e.g., [29]). In contrast, we used a motor task in which participants were provided with information that could help guide adjustments prior to the actual performance of the task. The involvement of pMFC in response to these types of cue thus may be contingent on the provision of useful, applicable information that can improve task performance. The ability to provide this kind of information is a particular benefit of our task design: it is much more difficult to do this in purely cognitive paradigms because in most cases it becomes impossible to prevent participants from starting to solve the task prior to the onset of an imperative signal.

Although the goal of our study was not to test the error-likelihood hypothesis directly, or to pit the effort-allocation hypothesis against the error-likelihood hypothesis, we would like to suggest that the involvement of pMFC in performance monitoring indexed here reflects the need for increased effort and not the concomitant increase in error likelihood. Although the present data cannot conclusively distinguish between these two possibilities, this interpretation finds support in two recent studies. The first showed that cues informing participants about the presence or absence of conflict in a subsequent Stroop-like task activated the pMFC independent of conflict and error-likelihood [21]. The second used a computational model along with neuroimaging data to dissociate conflict from error-likelihood by showing that the pMFC activity in a flanker task reflects the current level of cognitive demand rather than the likelihood of errors [62].

### Do conflict, errors and effort-related cues generate the same ERP component?

A central goal of our study was to compare the ERP component elicited by the High-Effort maze to the ERP components elicited by conflict and errors. Early definitions of ERP components were based on common latency, polarity and scalp distribution, but more recent views suggest that a more appropriate definition is based on common computational operations and neuro-anatomical generators [63]. The effort-allocation hypothesis postulates that errors, conflict and effort-related cues are all instances of a broad category of stimuli that indicate the need for increased effort to improve the performance of a task immediately or in the future. Accordingly, they could all trigger a common computational operation aimed at recruiting the resources necessary to achieve positive outcomes from immediate or future actions. The results from our neural source estimations showed that the pMFC was a common neural generator for all of the components analyzed, which is consistent with the idea that the components elicited by conflict, errors and effort-related cues arise from the same general neurocognitive process.

A potential problem with the idea that conflict, errors and effort-related cues elicited ERP components reflecting the same general neurocognitive processes is the fact that there were slight differences in the precise location of the pMFC activation foci for each component. Also, the additional foci that were activated in conjunction with the pMFC differed between the components. This subtle variation may reflect activation of physically distinct functional units in pMFC that contribute to a general cognitive function, but have dedicated anatomical connectivity with other brain areas [64]–[66]. The different functional units in the pMFC may be specialized but organized such that they act as a network that integrates information from different modalities to influence attention allocation, motor preparation and motor responses [67], [68]. Indeed, under different task demands it appears that the pMFC might modulate different task-related processing units. In auditory discrimination, more emphasis on speed and accuracy leads to pMFC activation and increases in early auditory potentials presumably by an up-modulation of task-relevant brain areas by the pMFC [69]–[71]. Similarly, in motor tasks the pMFC appears to up-modulate the gain of task-relevant units and down-modulate the gain of task-irrelevant units under situations requiring increased cognitive and physical effort [72], [73]. Thus, despite the differences in the activation foci that accompanied the pMFC activation, it is possible that the ERP components we analyzed all represent the activation of the pMFC to subserve a common general function, namely allocating the necessary effort to improve the chances of successful task performance.

### Effort allocation, action selection and exploratory behavior

Recent evidence has suggested that the role of the pMFC in allocating effort may be through a role in action selection involving effort-based cost-benefit analysis [7], [74]–[76]. In a T-maze paradigm where one arm of the maze is associated with high effort (i.e., climbing a barrier) and high reward, and the other arm with low effort and low reward, healthy rats consistently chose the high-effort / high-reward arm. However, after lesions to pMFC this pattern changes and they come to choose the arm with low effort and low reward. This deficit does not appear to be due to an inability to represent reward or to make the effort to climb the barrier. Rats return to choosing the high reward option if a second barrier is placed in the low effort arm, equating the effort required by each of the arms [7], [74]. It rather appears that pMFC lesions cause an inability to integrate the effort cost with the reward information such that the animal is unable to motivate performance of the high-effort task even when it has a favorable effort-to-reward ratio.

It is possible that in our experiment the increased pMFC activation in response to the High-Effort preview display at least partially reflects this role of the pMFC in integrating effort and reward to guide decisions about actions [32]. In the High-Effort condition, participants had to decide whether it was worthwhile to expend the effort necessary to successfully solve the task. In other words, they had to evaluate the effort to reward ratio and determine if it was favorable. This was not a decision of whether or not the participants should act during the High-Effort task, but instead a determination of whether the potential payoff justified the effort required to generate a reasonable chance of success.

The pMFC has also been associated with free choice during exploratory behavior [77]–[80] and this may have also played a role in the increased pMFC activation in response to the High-Effort maze. It appears that the pMFC is not involved simply in *holding* action-outcome contingencies that take into account the ratio of effort to reward, but is also involved in exploratory behavior *building* these relationships. Previous studies have shown that the pMFC is more strongly activated during early than late phases of learning [81]–[83]. Early in learning behavior is variable and involves the selection of potentially better actions on each trial. Late in learning exploitative behavior dominates and is expressed through the repetition of actions already known to produce the desired outcome. Most of the experimental paradigms used to test the role of the pMFC in decision-making and action selection have involved decisions between alternative options of ‘what to do’; for example pressing a button with the right or left hand. Outside of the laboratory, however, many if not most selections of action do not involve a decision of ‘what to do’ but rather a decision of ‘how to do it’. Most action goals can be achieved by many different actions or variants of the same action. In the present experiment, for example, participants did not have a choice of action, but could choose between different ways to produce the same action. One example was how participants decided to deal with the speed-accuracy tradeoff. Due to the time pressure, participants were forced to speed up their responses in the High-Effort task to avoid time-outs. However, the accuracy requirement of the task made it such that faster responses had a higher likelihood of leading to an error. Since participants did not achieve a high level of accuracy in the task during the experiment, they likely engaged in a trial-by-trial exploration of this speed-accuracy tradeoff to find the optimal way of solving the task. In the case of the Low-Effort task, participants maintained a high level of accuracy from early in the testing session, which presumably permitted them to simply exploit the same successful strategy by repetition. If the pMFC is indeed involved in guiding action-selection during exploratory behavior, this may have been reflected in the increased pMFC activation in response to the High-Effort maze.

## Conclusion

The present results show that a preview display indicating the need for increased effort activates the pMFC prior to the execution of the task. Under the same experimental paradigm, we also observed pMFC activation related to response conflict and errors in performance. We suggest that a unifying role for the pMFC in response preparation and performance monitoring might be to build and utilize action-outcome contingencies. These would be created based on effort-benefit analysis with the goal of supporting behaviors that lead to positive outcomes [32], [80], [84]. According to this view, the pMFC supports decisions to increase cognitive or physical effort by modulating the gain in task-related processing units in the brain [77], thereby engaging attention, cognition, and motor sequences that increase the likelihood of a positive outcome [31], [85]. This idea is well supported by patient studies showing that, in general, lesions to pMFC do not lead to deficits in error processing or conflict monitoring, but instead lead to apathy and failure to exert effort and control arousal states [86]–[88]. It is also consistent with the broad anatomical connections that pMFC has with motor, limbic and cognitive areas [89], [90]. The pMFC might thus be particularly fit to mediate the translation of information about past history of reinforcement and current environmental and internal states, triggering changes in cognition and behavior that ultimately improve the chances of positive performance outcomes.

## References

[1] Ridderinkhof KR, Ullsperger M, Crone EA, Nieuwenhuis S (2004) The role of the medial frontal cortex in cognitive control. Science 306(5695): 443–447. 15486290 10.1126/science.1100301

[2] Ullsperger M (2006) Performance monitoring in neurological and psychiatric patients. Int J Psychophysiol 59(1): 59–69. 16288812 10.1016/j.ijpsycho.2005.06.010

[3] Hadland KA, Rushworth MF, Gaffan D, Passingham RE (2003) The anterior cingulate and reward-guided selection of actions. J Neurophysiol 89(2): 1161–1164. 12574489 10.1152/jn.00634.2002

[4] Schweimer J, Saft S, Hauber W (2005) Involvement of catecholamine neurotransmission in the rat anterior cingulate in effort-related decision making. Behav Neurosci 119(6): 1687–1692. 16420173 10.1037/0735-7044.119.6.1687

[5] Shima K, Tanji J (1998) Role for cingulate motor area cells in voluntary movement selection based on reward. Science 282(5392): 1335–1338. 9812901 10.1126/science.282.5392.1335

[6] Thaler D, Chen YC, Nixon PD, Stern CE, Passingham RE (1995) The functions of the medial premotor cortex. I. Simple learned movements. Exp Brain Res 102(3): 445–460. 7737391 10.1007/BF00230649

[7] Walton ME, Bannerman DM, Alterescu K, Rushworth MF (2003) Functional specialization within medial frontal cortex of the anterior cingulate for evaluating effort-related decisions. J Neurosci 23(16): 6475–6479. 12878688 10.1523/JNEUROSCI.23-16-06475.2003 PMC6740644

[8] Cohen RA, Kaplan RF, Moser DJ, Jenkins MA, Wilkinson H (1999) Impairments of attention after cingulotomy. Neurology 53(4): 819–824. 10489048 10.1212/wnl.53.4.819

[9] Cohen RA, Kaplan RF, Zuffante P, Moser DJ, Jenkins MA, et al (1999) Alteration of intention and self-initiated action associated with bilateral anterior cingulotomy. J Neuropsychiatry Clin Neurosci 11(4): 444–453. 10570756 10.1176/jnp.11.4.444

[10] Ochsner KN, Kosslyn SM, Cosgrove GR, Cassem EH, Price BH, et al (2001) Deficits in visual cognition and attention following bilateral anterior cingulotomy. Neuropsychologia 39(3): 219–230. 11163601 10.1016/s0028-3932(00)00114-7

[11] Botvinick MM, Braver TS, Barch DM, Carter CS, Cohen JD (2001) Conflict monitoring and cognitive control. Psychol Rev 108(3): 624–652. 11488380 10.1037/0033-295x.108.3.624

[12] Botvinick MM, Braver TS, Yeung N, Ullsperger M, Carter CS, et al.. (2004) Conflict monitoring: Computational and empirical studies. In: Posner MI, editor. Cognitive neuroscience of attention. New York: Guilford Press. pp. 91–104.

[13] Botvinick MM, Cohen JD, Carter CS (2004) Conflict monitoring and anterior cingulate cortex: an update. Trends Cogn Sci 8(12): 539–546. 15556023 10.1016/j.tics.2004.10.003

[14] Carter CS, Braver TS, Barch DM, Botvinick MM, Noll D, et al (1998) Anterior cingulate cortex, error detection, and the online monitoring of performance. Science 280(5364): 747–749. 9563953 10.1126/science.280.5364.747

[15] Carter CS, Macdonald AM, Botvinick M, Ross LL, Stenger VA, et al (2000) Parsing executive processes: strategic vs. evaluative functions of the anterior cingulate cortex. Proc Natl Acad Sci U S A 97(4): 1944–1948. 10677559 10.1073/pnas.97.4.1944 PMC26541

[16] Yeung N, Cohen JD, Botvinick MM (2004) The neural basis of error detection: conflict monitoring and the error-related negativity. Psychol Rev 111(4): 931–959. 15482068 10.1037/0033-295x.111.4.939

[17] Falkenstein M, Hoormann J, Christ S, Hohnsbein J (2000) ERP components on reaction errors and their functional significance: a tutorial. Biol Psychol 51(2–3): 87–107. 10686361 10.1016/s0301-0511(99)00031-9

[18] Gehring WJ, Goss B, Coles MGH, Meyer DE, Donchin E (1993) A Neural System for Error-Detection and Compensation. Psychol Sci 4(6): 385–390.

[19] Holroyd CB, Coles MG (2002) The neural basis of human error processing: reinforcement learning, dopamine, and the error-related negativity. Psychol Rev 109(4): 679–709. 12374324 10.1037/0033-295X.109.4.679

[20] Miltner WHR, Braun C, Coles MGH (1997) Event-related brain potentials following incorrect feedback in a time estimation task: Evidence for a generic "neural system for error-detection". J Cogn Neurosci 9: 788–798. 23964600 10.1162/jocn.1997.9.6.788

[21] Aarts E, Roelofs A, van Turennout M (2008) Anticipatory activity in anterior cingulate cortex can be independent of conflict and error likelihood. J Neurosci 28(18): 4671–4678. 18448644 10.1523/JNEUROSCI.4400-07.2008 PMC6670453

[22] Alexander WH, Brown JW (2011) Medial prefrontal cortex as an action-outcome predictor. Nat Neurosci 14(10): 1338–1344. 21926982 10.1038/nn.2921 PMC3183374

[23] Brown JW, Braver TS (2005) Learned predictions of error likelihood in the anterior cingulate cortex. Science 307(5712): 1118–1121. 15718473 10.1126/science.1105783

[24] Brown JW, Braver TS (2007) Risk prediction and aversion by anterior cingulate cortex. Cogn Affect Behav Neurosci 7(4): 266–277. 18189000 10.3758/cabn.7.4.266

[25] Ide JS, Shenoy P, Angela JY, Chiang-shan RL (2013) Bayesian Prediction and Evaluation in the Anterior Cingulate Cortex. J Neurosci 33(5): 2039–2047. 23365241 10.1523/JNEUROSCI.2201-12.2013 PMC3711643

[26] Johnston K, Levin HM, Koval MJ, Everling S (2007) Top-down control-signal dynamics in anterior cingulate and prefrontal cortex neurons following task switching. Neuron 53(3): 453–462. 17270740 10.1016/j.neuron.2006.12.023

[27] Luks TL, Simpson GV, Feiwell RJ, Miller WL (2002) Evidence for anterior cingulate cortex involvement in monitoring preparatory attentional set. Neuroimage 17(2): 792–802. 12377154

[28] Weissman DH, Gopalakrishnan A, Hazlett CJ, Woldorff MG (2005) Dorsal anterior cingulate cortex resolves conflict from distracting stimuli by boosting attention toward relevant events. Cereb Cortex 15(2): 229–237. 15238434 10.1093/cercor/bhh125

[29] Nieuwenhuis S, Schweizer TS, Mars RB, Botvinick MM, Hajcak G (2007) Error-likelihood prediction in the medial frontal cortex: a critical evaluation. Cereb Cortex 17(7): 1570–1581. 16956979 10.1093/cercor/bhl068 PMC3752593

[30] Botvinick MM & Huffstetler S, McGuire JT (2009) Effort discounting in human nucleus accumbens. Cogn Affect Behav Neurosci 9(1): 16–27. 19246324 10.3758/CABN.9.1.16 PMC2744387

[31] Oliveira FTP, McDonald JJ, Goodman D (2007) Performance monitoring in the anterior cingulate is not all error related: expectancy deviation and the representation of action-outcome associations. J Cogn Neurosci 19(12): 1994–2004. 17892382 10.1162/jocn.2007.19.12.1994

[32] Rushworth MF, Walton ME, Kennerley SW, Bannerman DM (2004) Action sets and decisions in the medial frontal cortex. Trends Cogn Sci 8(9): 410–417. 15350242 10.1016/j.tics.2004.07.009

[33] Rushworth MF, Buckley MJ, Behrens TE, Walton ME, Bannerman DM (2007) Functional organization of the medial frontal cortex. Curr Opin Neurobiol 17(2): 220–227. 17350820 10.1016/j.conb.2007.03.001

[34] Braver TS, Barch DM, Gray JR, Molfese DL, Snyder A (2001) Anterior cingulate cortex and response conflict: effects of frequency, inhibition and errors. Cereb Cortex 11(9): 825–836. 11532888 10.1093/cercor/11.9.825

[35] Donkers FC, van Boxtel GJ (2004) The N2 in go/no-go tasks reflects conflict monitoring not response inhibition. Brain Cogn 56(2): 165–176. 15518933 10.1016/j.bandc.2004.04.005

[36] Nieuwenhuis S, Yeung N, van den Wildenberg W, Ridderinkhof KR (2003) Electrophysiological correlates of anterior cingulate function in a go/no-go task: effects of response conflict and trial type frequency. Cogn Affect Behav Neurosci 3(1): 17–26. 12822595 10.3758/cabn.3.1.17

[37] Kiehl KA, Liddle PF, Hopfinger JB (2000) Error processing and the rostral anterior cingulate: an event-related fMRI study. Psychophysiology 37(2): 216–223. 10731771

[38] Luu P, Tucker DM, Derryberry D, Reed M, Poulsen C (2003) Electrophysiological responses to errors and feedback in the process of action regulation. Psychol Sci 14(1): 47–53. 12564753 10.1111/1467-9280.01417

[39] Kerns JG, Cohen JD, MacDonald AW 3rd, Cho RY, Stenger VA, et al (2004) Anterior cingulate conflict monitoring and adjustments in control. Science 303(5660): 1023–1026. 14963333 10.1126/science.1089910

[40] Klein TA, Endrass T, Kathmann N, Neumann J, von Cramon DY, et al (2007) Neural correlates of error awareness. Neuroimage 34(4): 1774–1781. 17185003 10.1016/j.neuroimage.2006.11.014

[41] Delorme A, Makeig S (2004) EEGLAB: an open source toolbox for analysis of single-trial EEG dynamics including independent component analysis. J Neurosci Methods 134(1): 9–21. 15102499 10.1016/j.jneumeth.2003.10.009

[42] Gómez-Herrero G, De Clercq W, Anwar H, Kara O, Egiazarian K, et al.. (2006) Automatic removal of ocular artifacts in the EEG without a reference EOG channel. Proceedings of the 7th Nordic Signal Processing Symposium (NORSIG'2006). Reykjavik, Iceland. pp. 130–133.

[43] De Clercq W, Vergult A, Vanrumste B, Van Paesschen W, Van Huffel S (2006) Canonical correlation analysis applied to remove muscle artifacts from the electroencephalogram. IEEE Trans Biomed Eng 53(12): 2583–2587. 17153216 10.1109/TBME.2006.879459

[44] Hoechstetter K, Berg P, Scherg M (2010) BESA Research Tutorial 4: Distributed Source Imaging.

[45] Pascual-Marqui RD, Michel CM, Lehmann D (1994) Low resolution electromagnetic tomography: a new method for localizing electrical activity in the brain. Int J Psychophysiol 18(1): 49–65. 7876038 10.1016/0167-8760(84)90014-x

[46] Van Essen DC (2002) Windows on the brain: the emerging role of atlases and databases in neuroscience. Curr Opin Neurobiol 12(5): 574–579. 12367638 10.1016/s0959-4388(02)00361-6

[47] Grech R, Cassar T, Muscat J, Camilleri KP, Fabri SG, et al (2008) Review on solving the inverse problem in EEG source analysis. J Neuroeng Rehabil 5: 25. 18990257 10.1186/1743-0003-5-25 PMC2605581

[48] Van Veen V, Carter CS (2002) The timing of action-monitoring processes in the anterior cingulate cortex. J Cogn Neurosci 14(4): 593–602. 12126500 10.1162/08989290260045837

[49] Falkenstein M, Hoormann J, Hohnsbein J (1999) ERP components in Go/Nogo tasks and their relation to inhibition. Acta Psychol (Amst) 101(2): 267–291. 10344188 10.1016/s0001-6918(99)00008-6

[50] Falkenstein M, Hoormann J, Hohnsbein J (2002) Inhibition-related ERP components: Variation with modality, age, and time-on-task. J Psychophysiol 16(3): 167–175.

[51] Allain S, Carbonnell L, Falkenstein M, Burle B, Vidal F (2004) The modulation of the Ne-like wave on correct responses foreshadows errors. Neurosci Lett 372(1): 161–166. 15531109 10.1016/j.neulet.2004.09.036

[52] Mathalon DH, Whitfield SL, Ford JM (2003) Anatomy of an error: ERP and fMRI. Biol Psychol 64(1): 119–141. 14602358 10.1016/s0301-0511(03)00105-4

[53] Luu P, Tucker DM, Makeig S (2004) Frontal midline theta and the error-related negativity: neurophysiological mechanisms of action regulation. Clinical Neurophysiology 115(8): 1821–1835. 15261861 10.1016/j.clinph.2004.03.031

[54] Mathalon DH, Bennett A, Askari N, Gray EM, Rosenbloom MJ, et al (2003) Response-monitoring dysfunction in aging and Alzheimer's disease: an event-related potential study. Neurobiol Aging 24(5): 675–685. 12885575 10.1016/s0197-4580(02)00154-9

[55] Overbeek TJM, Nieuwenhuis S, Ridderinkhof KR (2005) Dissociable components of error processing: on the functional significance of the Pe vis-a-vis the ERN/Ne. J Psychophysiol 19(4): 319–329.

[56] O'Connell RG, Dockree PM, Bellgrove MA, Kelly SP, Hester R, et al (2007) The role of cingulate cortex in the detection of errors with and without awareness: a high-density electrical mapping study. Eur J Neurosci 25(8): 2571–2579. 17445253 10.1111/j.1460-9568.2007.05477.x

[57] Bunge SA, Ochsner KN, Desmond JE, Glover GH, Gabrieli JD (2001) Prefrontal regions involved in keeping information in and out of mind. Brain 124(10): 2074–2086. 11571223 10.1093/brain/124.10.2074

[58] Garavan H, Ross TJ, Murphy K, Roche RA, Stein EA (2002) Dissociable executive functions in the dynamic control of behavior: inhibition, error detection, and correction. Neuroimage 17(4): 1820–1829. 12498755 10.1006/nimg.2002.1326

[59] Konishi S, Nakajima K, Uchida I, Kikyo H, Kameyama M, et al (1999) Common inhibitory mechanism in human inferior prefrontal cortex revealed by event-related functional MRI. Brain 122(5): 981–991. 10355680 10.1093/brain/122.5.981

[60] Xue G, Aron AR, Poldrack RA (2008) Common neural substrates for inhibition of spoken and manual responses. Cereb Cortex 18(8): 1923–1932. 18245044 10.1093/cercor/bhm220

[61] Badgaiyan RD, Posner MI (1998) Mapping the cingulate cortex in response selection and monitoring. Neuroimage 7(3): 255–260. 9597666 10.1006/nimg.1998.0326

[62] Yeung N, Nieuwenhuis S (2009) Dissociating response conflict and error likelihood in anterior cingulate cortex. J Neurosci 29(46): 14506–14510. 19923284 10.1523/JNEUROSCI.3615-09.2009 PMC2831178

[63] Luck SJ (2004) Ten Simple Rules for Designing ERP Experiments. In Handy TC, editor. Event-related potentials: a methods handbook. MIT Press. pp. 17–32.

[64] Bush G, Luu P, Posner MI (2000) Cognitive and emotional influences in anterior cingulate cortex. Trends Cogn Sci 4(6): 215–222. 10827444 10.1016/s1364-6613(00)01483-2

[65] Naito E, Kinomura S, Geyer S, Kawashima R, Roland PE, et al (2000) Fast reaction to different sensory modalities activates common fields in the motor areas, but the anterior cingulate cortex is involved in the speed of reaction. J Neurophysiol 83(3): 1701–1709. 10712490 10.1152/jn.2000.83.3.1701

[66] Paus T, Petrides M, Evans AC, Meyer E (1993) Role of the human anterior cingulate cortex in the control of oculomotor, manual, and speech responses: a positron emission tomography study. J Neurophysiol 70(2): 453–469. 8410148 10.1152/jn.1993.70.2.453

[67] Bush G (2004) Multimodal studies of cingulate cortex. In Posner MI, editor. Cognitive neuroscience of attention. New York: Guilford Press. pp. 207–218.

[68] Bush G, Vogt BA, Holmes J, Dale AM, Greve D, et al (2002) Dorsal anterior cingulate cortex: a role in reward-based decision making. Proc Natl Acad Sci U S A 99(1): 523–528. 11756669 10.1073/pnas.012470999 PMC117593

[69] Mulert C, Leicht G, Pogarell O, Mergl R, Karch S, et al (2007) Auditory cortex and anterior cingulate cortex sources of the early evoked gamma-band response: relationship to task difficulty and mental effort. Neuropsychologia 45(10): 2294–2306. 17403530 10.1016/j.neuropsychologia.2007.02.020

[70] Mulert C, Menzinger E, Leicht G, Pogarell O, Hegerl U (2005) Evidence for a close relationship between conscious effort and anterior cingulate cortex activity. Int J Psychophysiol 56(1): 65–80. 15725491 10.1016/j.ijpsycho.2004.10.002

[71] Mulert C, Seifert C, Leicht G, Kirsch V, Ertl M, et al (2008) Single-trial coupling of EEG and fMRI reveals the involvement of early anterior cingulate cortex activation in effortful decision making. Neuroimage 42(1): 158–168. 18547820 10.1016/j.neuroimage.2008.04.236

[72] Isoda M, Hikosaka O (2007) Switching from automatic to controlled action by monkey medial frontal cortex. Nat Neurosci 10(2): 240–248. 17237780 10.1038/nn1830

[73] Taylor PC, Nobre AC, Rushworth MF (2007) Subsecond changes in top down control exerted by human medial frontal cortex during conflict and action selection: a combined transcranial magnetic stimulation electroencephalography study. J Neurosci 27(42): 11343–11353. 17942729 10.1523/JNEUROSCI.2877-07.2007 PMC6673042

[74] Walton ME, Bannerman DM, Rushworth MF (2002) The role of rat medial frontal cortex in effort-based decision making. J Neurosci 22(24): 10996–11003. 12486195 10.1523/JNEUROSCI.22-24-10996.2002 PMC6758435

[75] Walton ME, Kennerley SW, Bannerman DM, Phillips PE, Rushworth MF (2006) Weighing up the benefits of work: behavioral and neural analyses of effort-related decision making. Neural Netw 19(8): 1302–1314. 16949252 10.1016/j.neunet.2006.03.005 PMC2519033

[76] Croxson PL, Walton ME, O'Reilly JX, Behrens TE, Rushworth MF (2009) Effort-based cost–benefit valuation and the human brain. J Neurosci 29(14): 4531–4541. 19357278 10.1523/JNEUROSCI.4515-08.2009 PMC2954048

[77] Aston-Jones G, Cohen JD (2005) An integrative theory of locus coeruleus-norepinephrine function: adaptive gain and optimal performance. Annu Rev Neurosci 28: 403–450. 16022602 10.1146/annurev.neuro.28.061604.135709

[78] Cohen JD, McClure SM, Yu AJ (2007) Should I stay or should I go? How the human brain manages the trade-off between exploitation and exploration. Philos Trans R Soc Lond B Biol Sci 362(1481): 933–942. 17395573 10.1098/rstb.2007.2098 PMC2430007

[79] Quilodran R, Rothe M, Procyk E (2008) Behavioral shifts and action valuation in the anterior cingulate cortex. Neuron 57(2): 314–325. 18215627 10.1016/j.neuron.2007.11.031

[80] Rushworth MF (2008) Intention, choice, and the medial frontal cortex. Ann N Y Acad Sci 1124: 181–207. 18400931 10.1196/annals.1440.014

[81] Jueptner M, Stephan KM, Frith CD, Brooks DJ, Frackowiak RS, et al (1997) Anatomy of motor learning. I. Frontal cortex and attention to action. J Neurophysiol 77(3): 1313–1324. 9084599 10.1152/jn.1997.77.3.1313

[82] Milham MP, Banich MT, Claus ED, Cohen NJ (2003) Practice-related effects demonstrate complementary roles of anterior cingulate and prefrontal cortices in attentional control. Neuroimage 18(2): 483–493. 12595201 10.1016/s1053-8119(02)00050-2

[83] Raichle ME, Fiez JA, Videen TO, MacLeod AM, Pardo JV, et al (1994) Practice-related changes in human brain functional anatomy during nonmotor learning. Cereb Cortex 4(1): 8–26. 8180494 10.1093/cercor/4.1.8

[84] Shenhav A, Botvinick MM, Cohen JD (2013) The expected value of control: an integrative theory of anterior cingulate cortex function. Neuron 79(2): 217–240. 23889930 10.1016/j.neuron.2013.07.007 PMC3767969

[85] Hickey C, Chelazzi L, Theeuwes J (2010) Reward changes salience in human vision via the anterior cingulate. J Neurosci 30(33): 11096–11103. 20720117 10.1523/JNEUROSCI.1026-10.2010 PMC6633486

[86] Baird A, Dewar BK, Critchley H, Gilbert SJ, Dolan RJ, Cipolotti L (2006) Cognitive functioning after medial frontal lobe damage including the anterior cingulate cortex: a preliminary investigation. Brain Cogn 60(2): 166–175. 16384629 10.1016/j.bandc.2005.11.003

[87] Critchley HD, Mathias CJ, Josephs O, O'Doherty J, Zanini S, et al (2003) Human cingulate cortex and autonomic control: converging neuroimaging and clinical evidence. Brain 126(10): 2139–2152. 12821513 10.1093/brain/awg216

[88] Fellows LK, Farah MJ (2005) Is anterior cingulate cortex necessary for cognitive control? Brain 128(4): 788–796. 15705613 10.1093/brain/awh405

[89] Devinsky O, Morrell MJ, Vogt BA (1995) Contributions of anterior cingulate cortex to behaviour. Brain 118(1): 279–306. 7895011 10.1093/brain/118.1.279

[90] Paus T (2001) Primate anterior cingulate cortex: where motor control, drive and cognition interface. Nat Rev Neurosci 2(6): 417–424. 11389475 10.1038/35077500

